# Supplementary material for: Optimization of the Electrodeposition of Gold Nanoparticles for the Application of Highly Sensitive, Label-Free Biosensor
Source: Biosensors (Basel). 2019 Mar 31;9(2):50. doi: 10.3390/bios9020050 (PMC6628353; doi:10.3390/bios9020050)
Supplement: Supplementary file 1 [file biosensors-09-00050-s001.pdf]

biosensors

Supplementary Material

Optimization of the Electrodeposition of Gold Nanoparticles for the Application of Highly Sensitive, Label-Free Biosensor

Hao-Chun Chiang [1], Yanyan Wang [2], Qi Zhang [1] and Kalle Levon [1,*]

[1] Department of Chemical and Biomolecular Engineering, NYU Tandon School of Engineering, Six Metrotech Center, Brooklyn, NY 11201, USA; hcc293@nyu.edu (H.-C.C.); qz260@nyu.edu (Q.Z.)
[2] State Key Laboratory of Precision Measuring Technology & Instruments, School of Precision Instruments and Optoelectronics Engineering, Tianjin University, 300072 Tianjin, China; yanyanwang@tju.edu.cn
* Correspondence: kalle.levon@gmail.com

Section	Pages
Section S1. DOE Optimization of AuNPs Electrodeposition	2
Section S2. Measurement of AuNPs coverage	5
Section S3. Measurement of Gold Nanoparticle Size	8

DOE Optimization of Process Parameters for AuNPs Electrodeposition

Design of experiments (DOE) was performed by Model-Based Calibration Tool (The MathWorks, Inc.) One-stage test strategies were introduced for a single source of variations including (a) $HAuCl_4$ concentration, (b) H_2SO_4 concentration, (c) scan cycles, and (d) scan rate, which are the inputs of operating range in electrodeposition. 16 cases were performed, the operating parameters and data were collected in Table S1 (BSA). The output is the potentiometric measurement using open circuit potential technique also plotted in Figure 3. Gaussian process based modeling proved the adequacy of the developed model with root mean square error (RMSE) of 4.09, 4.4, and 5.2 for BSA, GOx, and Casein adsorption on AuNPs layer.

Table S1. The trials of electrodeposition process in the potentiometric response of BSA adsorption in design of experiments (DOE) analysis.

BSA	$HAuCl_4$ (mM)	H_2SO_4 (M)	Cycles	Scan Rate (mV/s)	DV
1	0.1	1	10	50	10
2	0.5	1	10	50	13
3	1	1	10	50	7.5
4	2	1	10	50	6
5	1	0.1	10	50	3
6	1	0.5	10	50	16
7	1	1	10	50	4
8	1	2	10	50	3
9	1	1	2	50	8
10	1	1	5	50	20
11	1	1	10	50	15
12	1	1	20	50	13
13	1	1	10	10	12.5
14	1	1	10	20	17
15	1	1	10	50	19
16	1	1	10	100	12

In the result of DOE analysis for BSA case, the contour plot of potentiometric response, interaction of $HAuCl_4$ concentration (mM) and H_2SO_4 concentration (M) us shown in Figure S1 and Figure S2. The optimal region of $HAuCl_4$ is the oval area of the center in 0.84 mM $HAuCl_4$ and 1M H_2SO_4. Likewise, the contour plot of potentiometric response, interaction of H_2SO_4 concentration (M) and scan cycle (times) which indicate the optimal region of electrodeposition condition with 1M H_2SO_4 and 12 cycles CV scans. The deviation of fitting result is shown in Figure S3, the predicted response agrees with experimental measurement when the V is less than 18 mV. The residual plot with predicted response value also indicates the good agreement of DOE analysis. After DOE calibration by above procedure for all three proteins, the optimal condition as well as predicted V could be found and list in Table S2. The optimal condition was selected within the optimal region suggested by DOE analysis as method 1, 2, and 3 for each BSA, GOx, and Casein respectively.

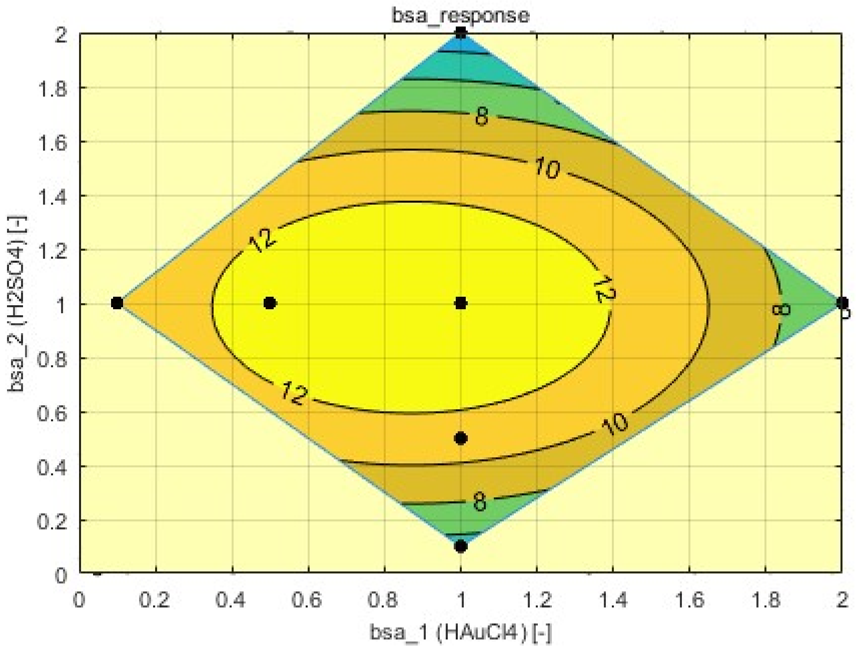

Figure S1. A contour interaction plot of potentiometric response, interaction of $HAuCl_4$ concentration (mM) and H_2SO_4 concentration (M).

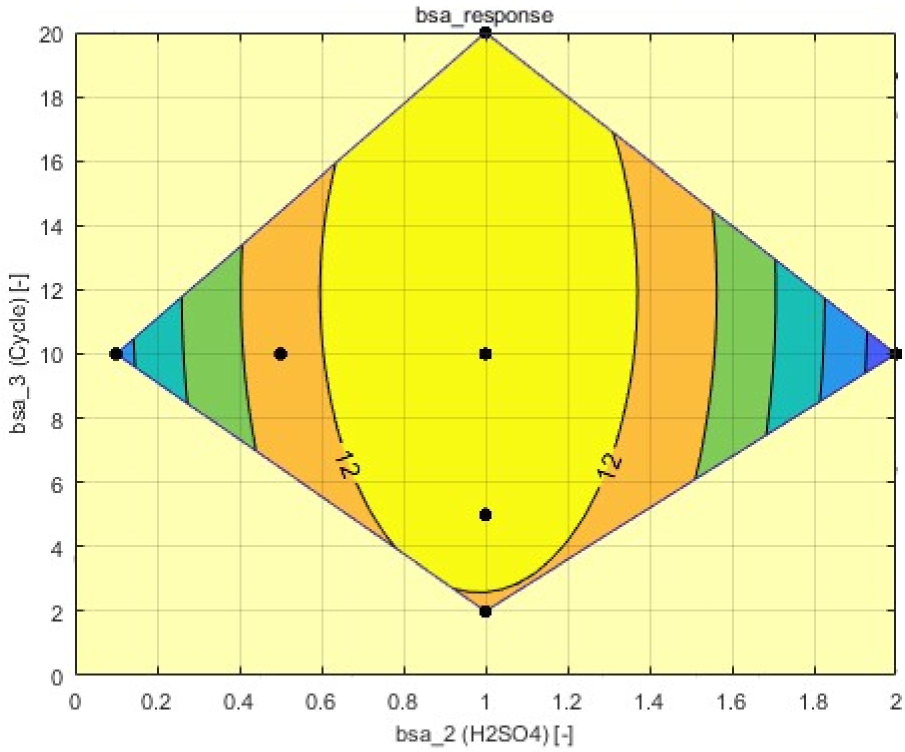

Figure S2. A contour interaction plot of potentiometric response, interaction of H_2SO_4 concentration (M) and cycle (times).

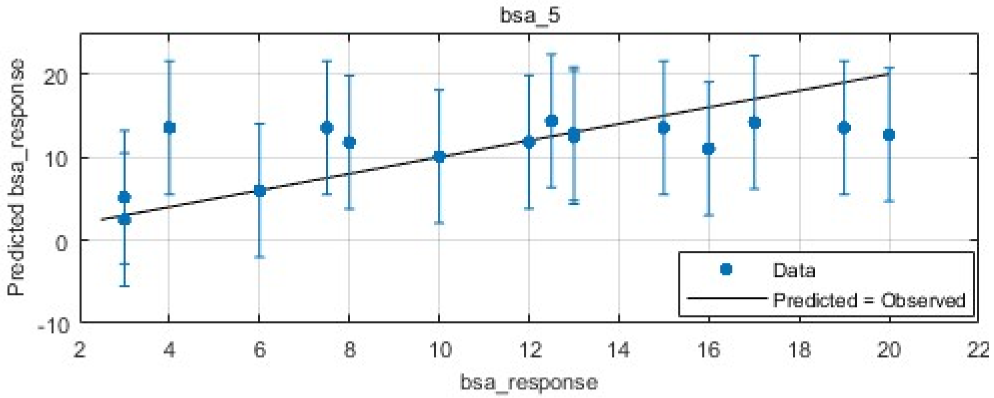

Figure S3. Relationship between predicted and experimental values of potentiometric response for BSA adsorption.

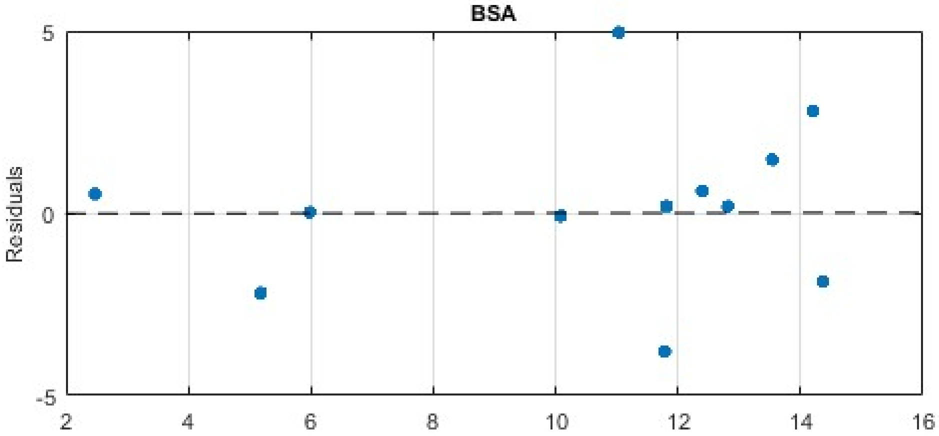

Figure S4. Normal probability plot of residuals vs. predicted (BSA).

Table S2. Design of the final parameter for the preparation of AuNPs layer.

Parameter/Protein	BSA	GOx	Casein
$HAuCl_4$	0.8	1	1.8
H_2SO_4	1	0.6	1
Cycles	12	5	7.5
Scan Rate	50	20	50
Predicted DV (mV)	13.4	14.1	16.5

Measurement of Particle Size

Analysis of the images was done using MATLAB (Mathworks, MA) and a custom written code. Connected Component Analysis algorithm is applied in particle size measurement [1,2], contiguous regions and discontiguous regions determination in binary image were returned using "regionprops" in MATLAB. The procedure of image processing as follow: The image was converted to a binary image using Otsu's thresholding. The closing process with disk structuring elements of 2 pixels was applied to remove noise included in the AFM image and enhance the edge. Each individual dot was then detected by using the Connected Component Analysis algorithm in the Image Processing Toolbox of MATLAB. The small unfilled objects of less than 4 pixels were subsequently removed. The filling process was required because the intensity of pixels surrounding the object was non-uniform. The sensitivity of Otsu's thresholding was optimized by maximizing particle size, however, when threshold is lowered to certain level, it would cause open circle within AuNPs and greatly reduce the mean particle size shown in Figure S5.

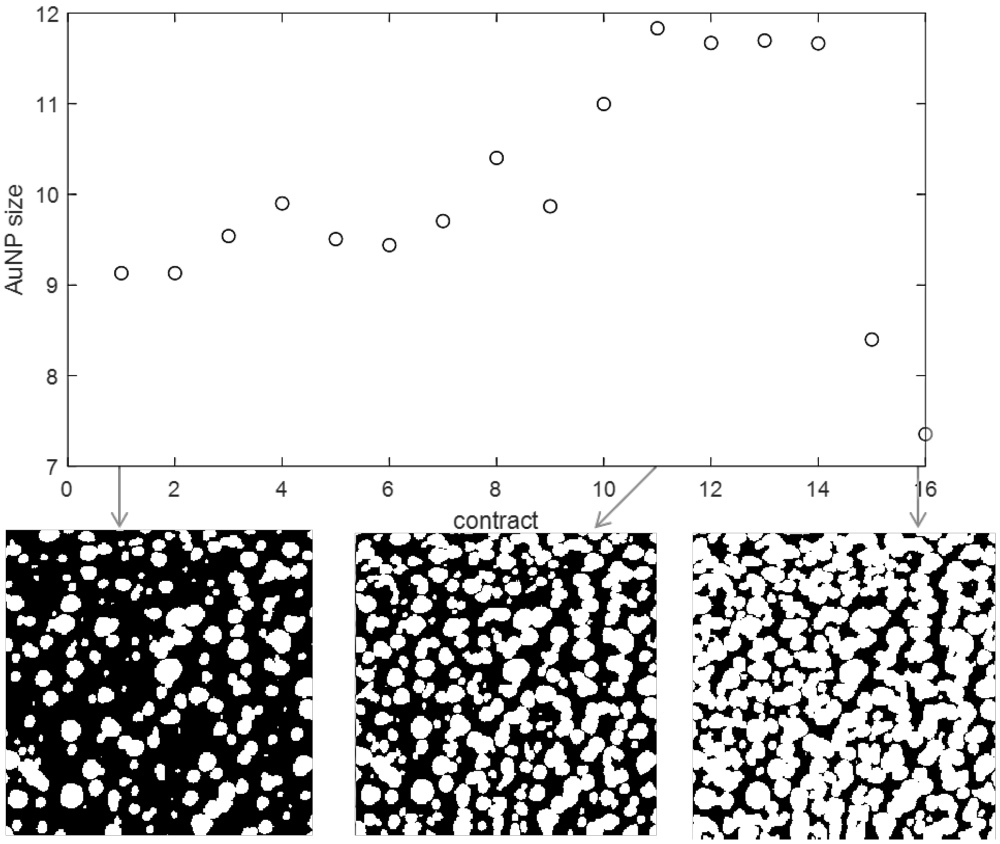

Figure S5. The optimization of sensitivity in connected component algorithm for particle measurement in AFM image, b in this example.

Collecting all size measurements for all the particles detected and measured with a track of around 400 repeat measurements in program provides a size distribution as shown in Figure S6. The mean size of AuNPs is 52.9 nm (6.77 pixel) for method 1. The actual particle size is converted according to AFM image scale. All the AFM images (except (d) due to low resolution) were processed in the same scheme and the results are shown in Table S3.

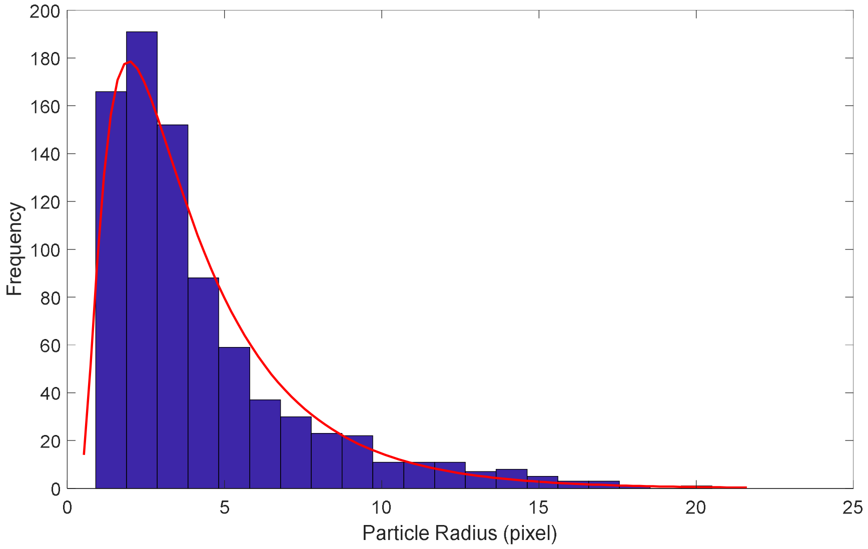

(**a**)

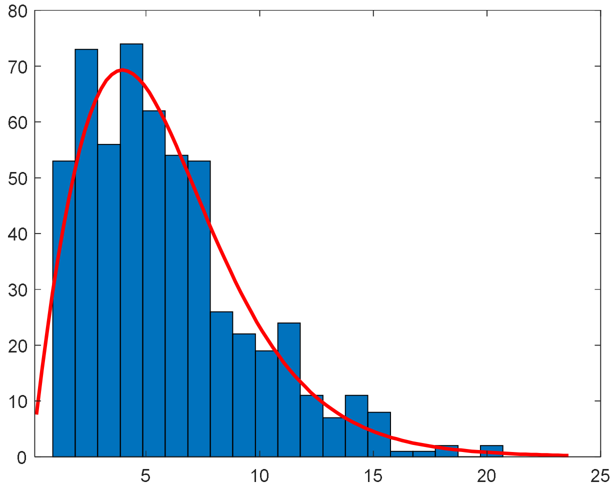

(**b**)

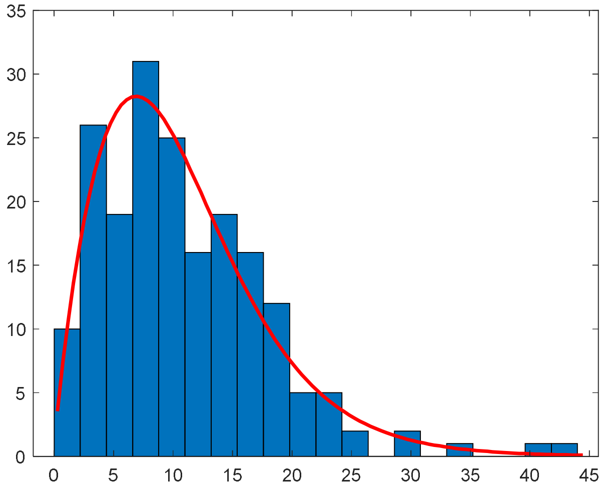

(**c**)

Figure S6. The distribution of nanoparticle of method 1 (**a**), method 2 (**b**), and method 3 (**c**) measured by connected component method and the fitting model is an asymmetric distribution.

Table S3. The gold nanoparticle size determined by image processing in AFM.

	Particle Size Radius	Diameter (pixel)	D (nm)
a	3.385	6.770	52.893
b	5.863	11.726	91.613
c	11.834	23.668	184.907
d	n.a.	-	-
e	4.987	9.975	77.927
f	12.666	25.332	197.907

Measurement of AuNPs Coverage

Bearing analysis characterizes the relative roughness of a surface in terms of high and low areas. The bearing ratio curve is the integral of the surface height histogram and the plots the total percentage of the surface above a reference plane as a function of the depth of that plane below the highest point in the image (Veeco Instruments Inc.). In the sample of analysis for image (c) illustrated in Figure S7, the color scale is corresponded to the depth of AFM probe, the minor peak in histogram figure indicate the surface of electrode uncovered by AuNPs which is 301.23 nm^2 and 97.251% coverage [3]. The same analytic scheme is also applied for other AFM image and the results shown in Table S4.

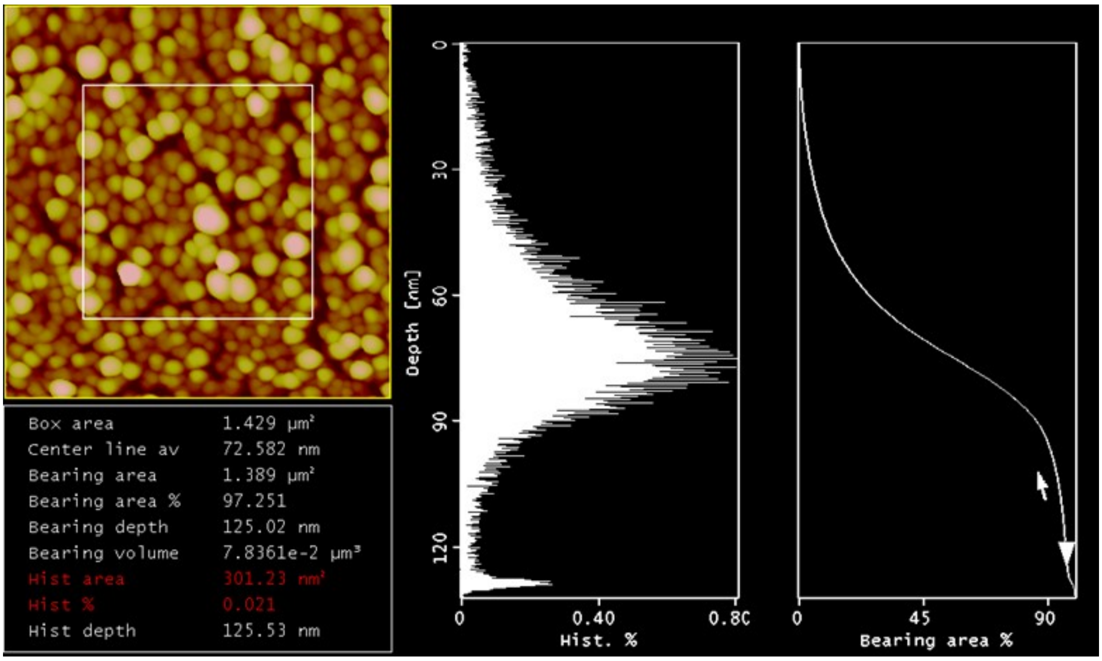

Figure S7. The bearing analysis in AFM image for AuNPs coverage.

Table S4. The AuNPs coverage.

	**D (nm)**	**Coverage (%)**
a	52.893	99.625
b	91.613	99.743
c	184.907	97.251
d	-	-
e	77.927	99.869
f	197.907	97.369

Reference

1. He, L.; Ren, X.; Gao, Q.; Zhao, X.; Yao, B.; Chao, Y. The connected-component labeling problem : A review of state-of-the-art algorithms. *Pattern Recognit.* **2017**, *70*, 25–43.
2. Wagner, T.; Lipinski, H.G. IJBlob : An ImageJ Library for Connected Component Analysis and Shape Analysis. *J. Open Res. Softw.* **2013**, *1*, e6.
3. Ballarin, B.; Cassani, M.C.; Scavetta, E.; Tonelli, D. Electrochimica Acta Self-assembled gold nanoparticles modified ITO electrodes : The monolayer binder molecule effect. *Electrochim. Acta* **2008**, *53*, 8034–8044.
